# Back to the Roots: Reconstructing Large and Complex Cranial Defects using an Image-based Statistical Shape Model

**DOI:** 10.1007/s10916-024-02066-y

**Published:** 2024-05-23

**Authors:** Jianning Li, David G. Ellis, Antonio Pepe, Christina Gsaxner, Michele R. Aizenberg, Jens Kleesiek, Jan Egger

**Affiliations:** 1 Institute for Artificial Intelligence in Medicine (IKIM), Essen University Hospital, Girardetstraße 2, 45131 Essen, Germany; 2 Department of Neurosurgery, University of Nebraska Medical Center, Omaha, NE 68198 USA; 3 Institute of Computer Graphics and Vision, Graz University of Technology, Inffeldgasse 16, Graz, 8010 Austria

**Keywords:** Statistical shape model, Deep learning, Domain shift, Generalization, Cranial implant design, Cranioplasty, Craniotomy, Craniectomy

## Abstract

Designing implants for large and complex cranial defects is a challenging task, even for professional designers. Current efforts on automating the design process focused mainly on convolutional neural networks (CNN), which have produced state-of-the-art results on reconstructing synthetic defects. However, existing CNN-based methods have been difficult to translate to clinical practice in cranioplasty, as their performance on large and complex cranial defects remains unsatisfactory. In this paper, we present a statistical shape model (SSM) built directly on the segmentation masks of the skulls represented as binary voxel occupancy grids and evaluate it on several cranial implant design datasets. Results show that, while CNN-based approaches outperform the SSM on synthetic defects, they are inferior to SSM when it comes to large, complex and real-world defects. Experienced neurosurgeons evaluate the implants generated by the SSM to be feasible for clinical use after minor manual corrections. Datasets and the SSM model are publicly available at https://github.com/Jianningli/ssm.

## Introduction

**Fig. 1:**
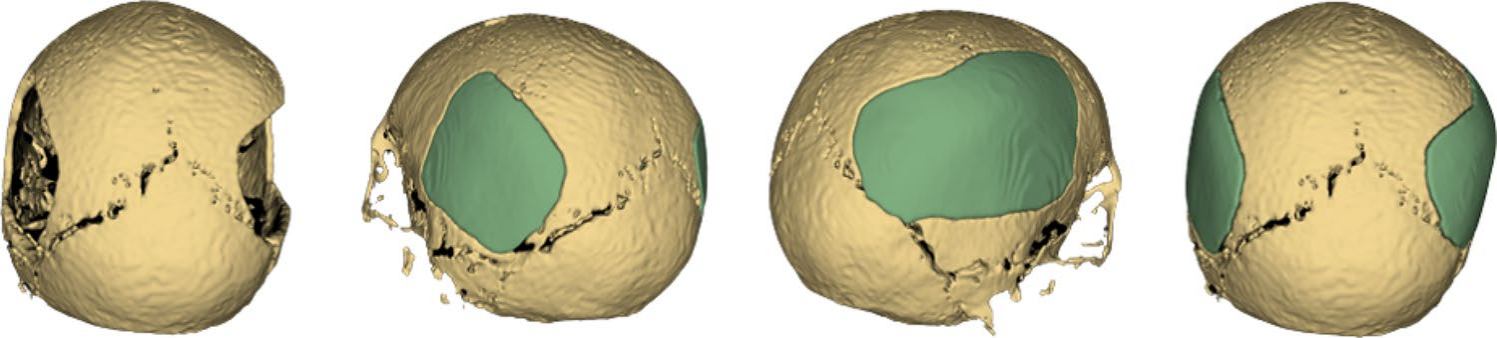
Teaser: reconstructing a skull with two defects. The skulls are shown in yellow and the implants in green

Before deep learning gained wide popularity [1], statistical shape model (SSM) and its variants (e.g., active shape models (ASMs) [2], active blobs [3] and active appearance models (AAMs) [4]) were broadly adopted in medical reconstruction and segmentation tasks, such as the reconstruction of craniofacial defects [5–10] and human rib cage [11], as well as the segmentation of hip joints [12] and organs [13]. In contrast to the latent shape features learned by deep neural nets, which are difficult to interpret, SSM offers the option to express a shape in an explicit manner, by linearly combining the mean shape and the principal modes of shape variations of a given shape pool. Surface meshes were common choices for anatomical shape representation in many SSM-based studies. Establishing dense point correspondence among the meshes is deemed the most demanding part in building an SSM, especially when the medical images, from which the meshes are derived, are of high resolution [14]. Methods that establish point correspondence automatically are typically based on a mesh-to-mesh registration procedure (e.g., Iterative Closest Point [15]), where the meshes are registered to a reference mesh through a similarity transformation (scaling, rotation and translation). However, popular state-of-the-art segmentation methods and various medical applications are image-based [16–20]. It is therefore desirable to circumvent the image-to-mesh conversion procedure and build an SSM directly on volumetric images [21–23]. Automatic cranial implant design is another typical application that uses images as the initial shape representation i.e., binary voxel occupancy grid [24]. Existing deep learning-based methods usually train a deep neural net on hundreds of skull images with either synthetic defects [25–28] or real-world clinical defects [29], which are however often not publicly available or large enough for training deep models. These approaches are data- and computation- intensive, and most importantly, the reconstruction quality for large and complex defects remains inadequate for clinical use [30, 31]. The failure can largely be attributed to distribution shift of the defects: the synthetic defects in the training set have different distribution from that of the test set. Augmenting the training set intensively has proven to be an effective solution to the distribution adaptation problem [32]. However, current development in data augmentation-enhanced deep learning is still evaluated as substandard by experienced neurosurgeons [30, 32]. A method that is insensitive to the defects may potentially avoid the distribution shift problem in cranial defect reconstruction. To this end, we propose an SSM-based method for automatic cranial implant design that relies only on complete skulls. Unlike previous mesh-based SSM for craniofacial defect reconstruction [6, 7, 9], our SSM is built directly on volumetric skull images represented as binary voxel grids. We show that the dense point correspondence for mesh-based SSM can be approximated through an image registration and warping step among the voxel grids, and that the mean shape and shape variations of the skulls can be calculated thereafter. Besides the expected robustness against large and complex cranial defects, another favorable property of an SSM-based method is that the skull shapes can be expressed explicitly, unlike deep learning-based approaches that often learn an uninterpretable shape representation. The proposed SSM is evaluated on both synthetic defects and irregularly shaped clinical defects from three cranial implant design tasks. We show that even though deep learning still beats SSM on synthetic defect reconstruction, its performance is inferior when it comes to large and complex clinical defects (Fig. 1).

## Method

### Overview

The main workload of building an SSM lies in finding the mean shape $\bar{S}$ and the primary shape variations $\mathbf {\phi }$ of a given shape pool, as specified by Eq. (1):

1 $$\begin{aligned} S=\bar{S}+\sum _{i=1}^{C}\lambda _i\mathbf {\phi }_i \end{aligned}$$

$\lambda _i$ is the weight of variation $\mathbf {\phi }_i$. *C* is the number of shape variations chosen for reconstructing the new shape *S*. Let $\textbf{X}$ be a shape pool containing *C* binary volumetric images $\textbf{x}_i \in \{0,1\}^{D_i}$, in which the $j_{th}$ image $\textbf{x}_j$ ($0<i, j\leqslant C$) is selected as a reference. $D_i$ is the image dimension. The non-zero voxels in these images constitute the geometry of the shapes and are regarded by default as the shape *landmarks*. Therefore, establishing the point correspondences between all the images in the shape pool can be achieved by simply registering $\textbf{x}_i$ ($i\ne j$) to the reference image $\textbf{x}_j$:

2 $$\begin{aligned} \textbf{Tr}: x_i\rightarrow x_j \end{aligned}$$

$\textbf{Tr}$ is a transformation that warps the images in $\textbf{X}$ into the space of $\textbf{x}_j$: $x'_i=\textbf{T} (x_i)$, $x'_i \in R^{D_j}$. Let $$X'=\begin{Bmatrix} x'_i \,| \, i\in Z, 0< i\leqslant C \end{Bmatrix}$$, $\mathbf {X'} \in R^{ D_j \times C}$ be the set of warped shapes. The mean shape $\bar{S} \in R^{D_j}$ of $\textbf{X}'$ is calculated as:

3 $$\begin{aligned} \bar{S}=\frac{1}{C}\sum _{i}^{C}x'_i \end{aligned}$$

To extract the shape variations $\mathbf {\phi }_i \in R^{D_j}$, principal component analysis (PCA) [33] is used. Let $$\mathbf {\Phi }=\begin{Bmatrix}\phi _i\,|\,i\in Z, 0< i\leqslant C\end{Bmatrix}$$, $\mathbf {\Phi } \in R^{C \times D_j}$ ($C\ll D_j$) be the set of chosen shape variations. Transforming $X'$ into the PCA space can be achieved via:

4 $$\begin{aligned} \mathbf {\Phi }\cdot X'=X_{pca}' \end{aligned}$$

$X_{pca}' \in R^{C \times C}$. The $X_{pca}'$ is given by the PCA function from the *scikit-learn* package and $X'^{-1}$ is computed from the training set *X*. Therefore, we can calculate the variation matrix $\mathbf {\Phi }$ as follows^1^:

5 $$\begin{aligned} \mathbf {\Phi } =X_{pca}'\cdot X'^{-1} \end{aligned}$$

$X'^{-1}$ is a pseudo inverse of $X'$. Given a test shape *y*, it is first registered to the reference image $y'=\textbf{Tr}(y), y' \in R^{D_j}$ and then mapped into the PCA space defined by the shape pool *X*:

6 $$\begin{aligned} \lambda =\mathbf {\Phi } \cdot y' \end{aligned}$$

$$\lambda =\begin{Bmatrix}\lambda _i \,|\,i\in Z, 0< i\leqslant C\end{Bmatrix}$$. We rescale $\lambda _i$ to [0,1] via: $(\lambda _i -min(\lambda ))/max(\lambda )-min(\lambda )$. Given $\lambda$, $\mathbf {\Phi }$ and $\bar{S}$, the new shape can be computed according to Eq. (1). In our work, $\textbf{Tr}$ is chosen to be a similarity transformation. The reconstructed shapes can be warped to their original space via an inverse transformation $\textbf{Tr}^{-1}$.

### Volumetric Shape Completion

#### Shape Warping

An intuitive way to complete a defective shape *y* is to warp it to the space of a complete shape $x_j$, which can be achieved through a registration process (i.e., $y'=\textbf{Tr}(y)$). Since the anatomical landmarks of the two shapes are aligned because of registration, a following subtraction operation^2^ between the two shapes can yield the missing portion of the defective shape $y_m$:

7 $$\begin{aligned} y_m = x_j - y' \end{aligned}$$

The addition of $y'$ and $y_m$ produces the complete shape corresponding to $y'$. By inverting the registration, we can obtain the complete shape $y_c$ corresponding to *y* in its original space:

8 $$\begin{aligned} y_c=\textbf{Tr}^{-1}(y_m + y') \end{aligned}$$

The concept is similar to that of a template-based shape completion approach [34], in which the missing part of a defective shape is taken from a complete template shape. The choice of the template shape affects the authenticity of $y_m$. Optimally, a shape $x_j$ that is general and representative of the shape class should be chosen as the template, to ensure that the registration error between $x_j$ and $y'$ is small and the missing part is taken from anatomically close regions on the template. The template shape can be from a single image like $x_j$, or the mean shape $\bar{S}$ of a shape pool, as specified in Eq. (3).

#### SSM for Volumetric Shape Completion

If the shape pool *X* consists of complete shapes, while the test shape *y* refers to a defective shape, applying Eqs. (1)–(6) would give the complete counterpart corresponding to *y*. In this sense, SSM can be used for shape completion tasks. In [35], the authors used PCA for skull shape completion and showed that, by applying PCA and an inverse PCA consecutively to a defective skull, a complete skull can be obtained. Equations (1) and (6) give the mathematical explanation: the PCA computes the skull shape variations $\mathbf {\Phi }$ from the training samples and the weights $\lambda$ from the warped defective skull $y'$, while the inverse PCA, according to the the implementation of *inverse_transform* from the *scikit-learn* package, computes:

9 $$\begin{aligned} S=\bar{S} +\lambda \cdot \mathbf {\Phi } \end{aligned}$$

which is equivalent to Eq. (1). Incorporating Eq. (6) into Eq. (9) we get:

10 $$\begin{aligned} S=\bar{S} + \mathbf {\Phi }\cdot y'\cdot \mathbf {\Phi }=\bar{S}+y'\cdot \mathbf {\Phi }^T\cdot \mathbf {\Phi } \end{aligned}$$

In both [35] and Eq. (1), the principal components of a defective skull are used as the weights of the shape variations. An obvious shortcoming is that, if the defects are too large, the principal components computed from a defective skull might not reflect the true distribution of the shape variations of a complete skull. For example, given a defective skull whose facial bone far outweighs the cranium due to a large cranial defect, the weight $\lambda _{k}$ of the variation concerning the facial area $\phi _{k}$ ($0<k\leqslant C$) would overwhelm the other variations, resulting in an inappropriate reconstruction of the region of interest (ROI, i.e., the cranium) for the cranial implant design task.

## Experiment and Results

### Dataset and Metrics

We evaluated our method on three datasets: the 11 clinical cases of defective skulls from Tasks 2 of the AutoImplant II challenge [36],^3^ the 29 craniotomy skulls from MUG500+ [37], and the 110 test skulls with synthetic defects from Task 3 of AutoImplant II. To conform to the evaluation scheme of the AutoImplant II challenge, we measure the reconstruction accuracy using dice similarity coefficient (DSC), border DSC and 95 percentile Hausdorff distance (HD95). The complete skulls from the training set of Task 3 were used as the shape pool *X*. The image dimension is $D_i= 512\times 512 \times Z_i$^4^ ($Z_i$ differs for different images). As calculating $\mathbf {\Phi }$ (Eqs. (4) and (5)) from high resolution images is a computationally expensive process, we only used $C=30$ (out of 100) complete skulls for experiments involving $\mathbf {\Phi }$. The reference skull $x_j$ is chosen to be case *001.nrrd* in the Task 3 training set and $Z_j=222$. All the training and test samples are registered to *001.nrrd* through a similarity transformation $\textbf{Tr}$.

For the synthetic defect reconstruction task (“Reconstruction of Synthetic Cranial Defects” section), four deep learning-based approaches, i.e., [27, 32, 38, 39] are chosen for a comparison study. For Task 2 (“Task2 of the AutoImplant II Challenge” section), two deep learning-based approaches are used for comparison [31, 32]. The networks used in these approaches are based on an auto-encoder [27, 39] or U-Net [31, 32, 38]. In [32, 38], the authors adopted a registration-based data augmentation scheme that substantially increased the number of training samples, and trained a 3D U-Net on the augmented dataset. Their solutions won the first place in the AutoImplant challenge. In [27, 39], the authors used a standard 3D auto-encoder trained on the original challenge dataset without augmentation. In [31], a two-step learning process was employed, wherein one 3D U-Net learns the shape of the implant, while a subsequent 3D U-Net refines the learned implant.

### Reconstruction of Synthetic Cranial Defects

**Fig. 2:**
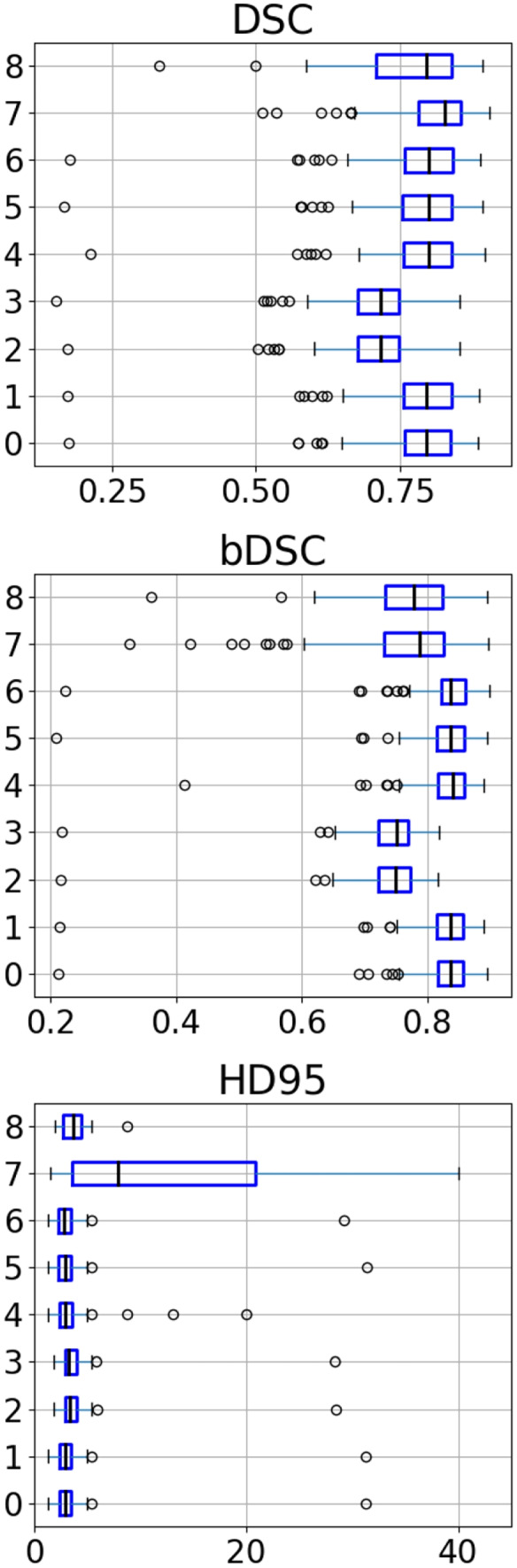
Boxplots of DSC, bDSC and HD95. **0**: SSM (30), **1**: SSM (30) + DL, **2**: $\sum _{i=1}^{d_0}\lambda _i\Phi _i$ ($\lambda _i=1$), **3**: $\sum _{i=1}^{d_0}\lambda _i\Phi _i$, **4**: $\bar{S}$ (50), **5**: $\bar{S}$ (30), **6**: $x_j$, **7**: DL, **8**: [35]

The 110 test skulls from Task 3 contain synthetic defects. In this experiment, we evaluate different methods for creating a skull template for shape warping: averaging 30 complete skulls (denoted as $\bar{S}$ (30)), averaging 50 complete skulls (denoted as $\bar{S}$ (50)) and using only a single skull (denoted as $x_j$). The 30 skulls are a subset of the 50 skulls. The results are compared with two deep learning-based methods from [27, 39].^5^ Besides, shape reconstruction using only the shape variations is also evaluated: $\sum _{i=1}^{d_0}\lambda _i\Phi _i$ ($\lambda _i=1$) and $\sum _{i=1}^{d_0}\lambda _i\Phi _i$. For the former, the $\lambda _i$ is set to one. For the latter, $\lambda _i$ is calculated based on Eq. (6). The DSC, bDSC and HD95 for these shape completion methods are reported in Table 1 and Fig. 2.

**Table 1:** Quantitative results (mean DSC, bDSC and HD95) on the 110 test cases of Task 3

Methods $\setminus$ Scores	DSC	bDSC	HD95 (mm)
$\bar{S}$ (30)	0.7840	0.8265	3.1989
$\bar{S}$ (50)	0.7853	0.8287	3.2447
$x_j$	0.7854	0.8285	3.1700
SSM (30)	0.7832	0.8255	3.2157
SSM (30) + DL	0.7830	0.8253	3.2170
[27, 39]	0.8058	0.7638	13.2891
$\sum _{i=1}^{d_0}\lambda _i\Phi _i$ ($\lambda _i=1$)	0.7054	0.7403	3.6783
$\sum _{i=1}^{d_0}\lambda _i\Phi _i$	0.7064	0.7411	3.6601
L. Yu. et. al. [35]	0.7728	0.7716	3.6803
D. G. Ellis, et.al. [38]	0.9440	-	-
M. Wodzinski et. al. [32]	0.9329	0.9530	1.4781

**Fig. 3:**
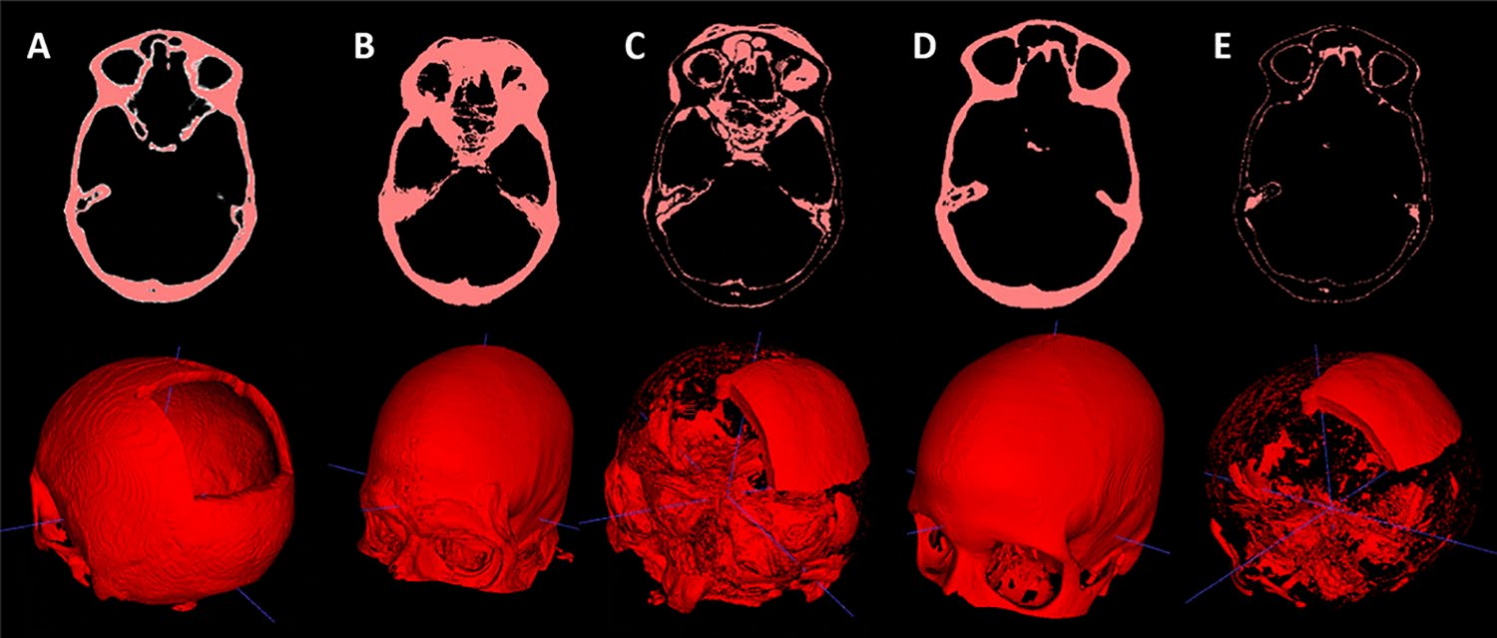
**A**: the input defective skull. **B**: the mean skull ($\bar{S}$ (30)). **C**: the subtraction between **B** and **A**. **D**: the reference skull $x_j$. E: the subtraction between **D** and **A**

The results bear important implications: **(1)** Using a single skull image $x_j$ as the template for shape warping can produce reasonable results, qualitatively and quantitatively (third row, Table 1, and Fig. 3E). However, it should be noted that the conclusion applies only to this specific task, where the region of interest (ROI), i.e., the cranium, is structurally simple. Using a single image as the template would likely fail on more complex facial structures. **(2)** Shape template derived from a single shape ($x_j$) produces comparable results to that from a mean shape averaged from 30 ($\bar{S}$ (30)) or 50 ($\bar{S}$ (50)) images. Figure 3 gives an example of the results obtained using shape warping. We can see that $\bar{S} (30)$ (Fig. 3B) shows no noticeable difference on the cranium compared to $x_j$ (Fig. 3D). As a result, subtracting the input from the templates (Eq. (7)) produces similar implants. The main difference lies in the facial area and the interior subtle structures (Fig. 3C and E). The reconstruction accuracy depends largely on how well the target matches with the template on the ROI (e.g., cranium or facial bones) during the warping and registration process. It is relatively easier to register different craniums than the facial structures from different subjects. In a facial reconstruction task, using a mean facial shape (e.g., Fig. 3B) would potentially produce more accurate reconstruction compared to using a single image. **(3)** In comparison to the deep learning-based approaches [32, 38], the shape warping- and SSM-based methods achieve inferior results on synthetic defects quantitatively. However, it should be noted that both [38] and [32] used an intensively augmented dataset during training, while only 30 images were used to build the SSM.

### MUG500+

**Fig. 4:**
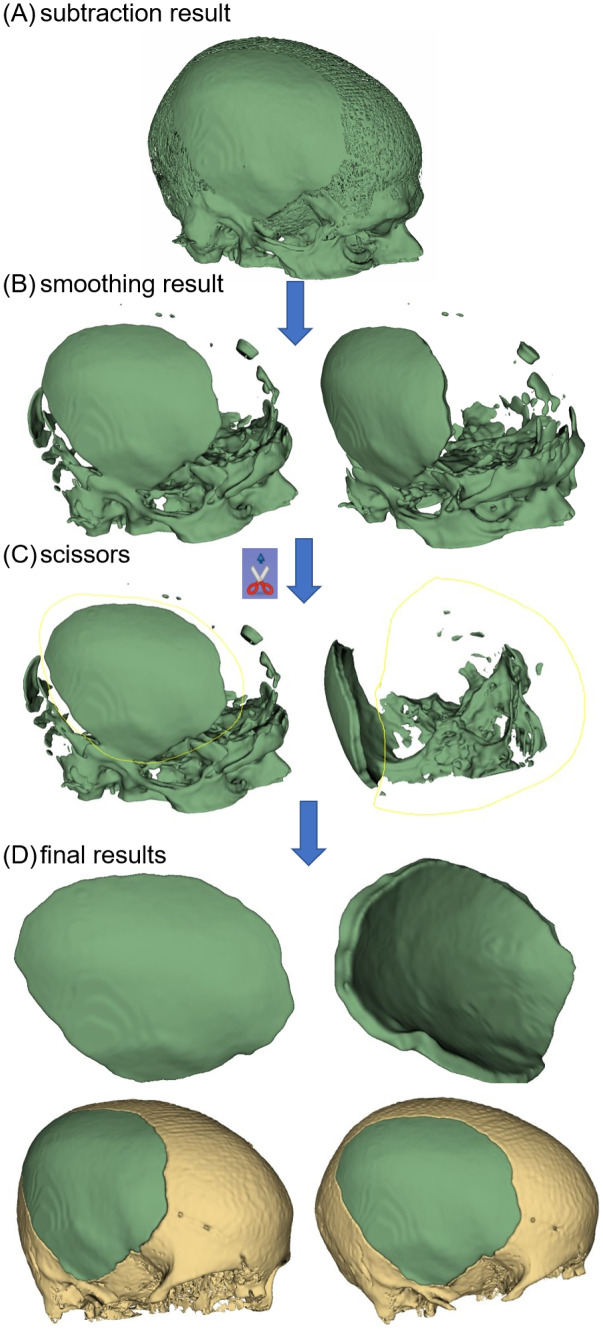
Manually extract the implants from the subtraction results using 3D Slicer. **A** The subtraction result. **B**-**D** The post-processing results of an implant shown in two different views. The last row shows how the final implant aligns with the skull

**Fig. 5:**
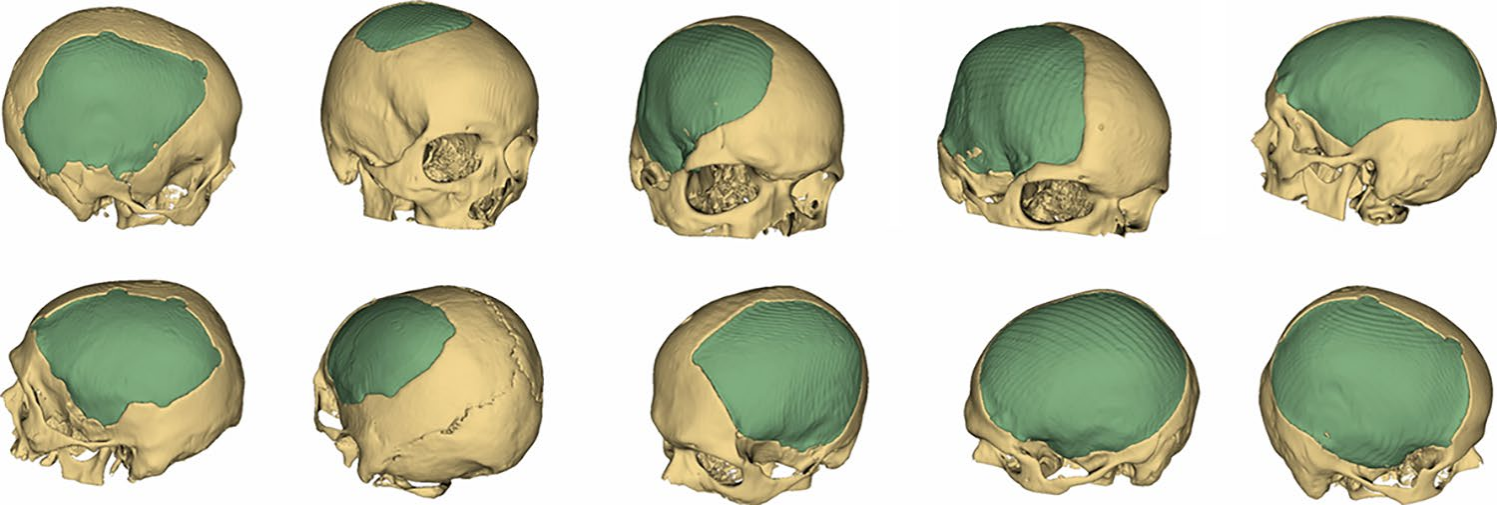
Exemplary results (from $\bar{S}$ (50)) on the craniotomy skulls from the MUG500+ dataset. The 29 generated implants can be downloaded at https://doi.org/10.6084/m9.figshare.19328816.v3

This section presents the implant generation results on the craniotomy skulls from the MUG500+ dataset [37]. Figure 4 shows the 3D Slicer-based manual processing procedure of an implant (Fig. 4(A)) generated by subtracting the input defective skull from the skull reconstructed by SSM (30). First, a median smoothing filter is applied to the subtraction result to partially disconnect the implant from the noise (Fig. 4B). The smoothing kernel size should be chosen individually based on the specific case. Second, the *scissors* functionality is used to erase the delineated area (Fig. 4C) to fully remove the noise and extract the implant. Figure 4(D) shows the final implant. Step (B) and (C) are done manually using 3D Slicer (https://www.slicer.org/) [40]. Alternatively, the implant can be extracted automatically by applying morphological opening and connected component analysis consecutively to the subtraction result. However, it is recommended to follow the manual post-processing workflow in Fig. 4 for an optimal outcome. Figure 5 presents the automatically generated implants for some large and complex defects in the MUG500+ dataset. The implants are generated by $\bar{S}$ (50) and manually post-processed according to Fig. 4. We can see that some of the defects are large, extending across almost half of the cranium and having rather irregular shapes. Nonetheless, the defects are still satisfactorily reconstructed. Notably, Fig. 1 (the teaser image) shows that the method is still effective when multiple large defects exit on the craniotomy skull. The completed skulls obtained according to Eq. (8) preserve the anatomical aesthetics of normal human skulls. The MUG500+ dataset also provides manually designed implants (i.e., surface models in *.stl* format) for the 29 craniotomy skulls. We convert the manual designs to images (*.nrrd*) for a quantitative comparison with our SSM-based methods. The results are given in Table 2. Since our work is the first to use the craniotomy dataset for evaluation, results from deep learning-based approaches are not available. Therefore, we only calculate and report the results from $\bar{S}$ (50). Note that the quantitative scores should be interpreted with care, as they only reflect how well the reconstructions match with the manual designs rather than their actual clinical feasibility [30].

**Table 2:** Quantitative results (produced by $\bar{S}$ (50)) for the MUG500+ craniotomy dataset

Methods $\setminus$ Scores	DSC	bDSC	HD95
$\bar{S}$ (50)	0.5471	0.5761	5.0000

### Task2 of the AutoImplant II Challenge

**Fig. 6:**
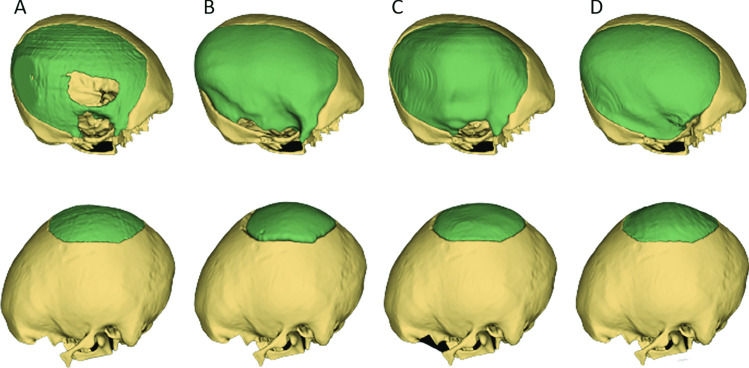
Qualitative comparison between different implant design methods on Task 2@AutoImplant II. **A** [32], **B** [35], **C** [31], **D** Ours. The 11 generated implants can be downloaded at https://doi.org/10.6084/m9.figshare.19328816.v3

We also apply the SSM-based method on the 11 clinical defective skulls from Task 2 of the AutoImplant II challenge. As described by Ellis et al. [30], the implant designs were quantitatively compared to reconstructions from postoperative imaging of the actual implant the patients have received for treatment. Table 3 shows these quantitative results from $\bar{S}$ (50) and SSM (30), as well as from the AutoImplant II submissions. The $\bar{S}$ (50) and SSM (30) had the best Hausdorff 95 scores than all other submissions but scored worse than some other submissions in the dice similarity and boundary dice similarity scores. Figure 6 shows a qualitative comparison. The first row in Fig. 6 shows the reconstruction results for a large defect with complex lower borders that extend to the zygomatic bone. We can see that deep learning-based approaches either produce incomplete reconstruction (Fig. 6A) or fail to cover the complex lower borders (Fig. 6B, C). In contrast, our method shows improved reconstruction in terms of completeness and border consistency (Fig. 6D). An advanced registration method that accurately aligns the corresponding anatomical landmarks between the template and target skull or a surface extrapolation method that guarantees smooth surface transition, as presented in [41, 42], is required to further improve border consistency, especially when it comes to complex and irregular defects.

**Table 3:** Quantitative results for Task 2 of the AutoImplant II challenge

Methods $\setminus$ Scores	DSC	bDSC	HD95
$\bar{S}$ (50)	0.5007	0.4449	8.2539
SSM (30)	0.5055	0.4470	7.9042
Wodzinski et al. [32]	0.5241	0.4823	54.5165
Yu et al. [35]	0.5118	0.4547	8.3486
Mahdi et al. [31]	0.3028	0.3092	71.4193

However, comparing the implant designs to the reconstructions of the implants from the postoperative CT imaging do not necessarily serve as a reliable metric for the quality of the implant designs [30]. For this reason, the implant designs were also qualitatively evaluated by an experienced neurosurgeon. The implant designs were judged based on completeness, false positive area, fit, and overall feasibility as described by Ellis et al. [30]. As shown in Table 4, the $\bar{S}$ (50) implant designs had better overall feasibility, better fit, and less false positive area than the submissions from the Autoimplant II challenge. While none of the submissions from the Autoimplant II challenge were deemed feasible without modifications, 4 out of 11 of the $\bar{S}$ (50) designs were deemed feasible with only minor flaws. Therefore, the $\bar{S}$ (50) designs represent a substantial improvement in the clinical feasibility of implant designs compared to the deep learning-based challenge submissions. The main issues of the implant designs from $\bar{S}$ (50) were that they did not always extend far enough in the superior direction to fully restore the natural skull shape and that the implants were often too thick.

**Table 4:** Qualitative evaluation scores for Task 2 of the AutoImplant II challenge by neurosurgeon MRA. The scores have been normalized such that 0 is the lowest possible score and 1 is the highest possible score. Completeness (Comp) evaluates the amount of the defect that the implant design covers. False positive area (FPA) evaluates the amount of amount of implant design outside of the defect area. Fit evaluates the shape of the implant design relative to the defect. Feasibility evaluates whether the implant design could be used in surgery. See Ellis et al. for the qualitative analysis methods [30]

Methods $\setminus$ Scores	Comp	FPA	Fit	Feasibility
$\bar{S}$ (50)	0.89	0.73	0.64	0.62
Wodzinski et al. [32]	0.93	0.57	0.55	0.42
Yu et al. [35]	0.80	0.59	0.36	0.42
Mahdi et al. [31]	0.76	0.43	0.45	0.33

## Discussion

In automatic cranial implant design, deep learning-based approaches that rely on a *defect-complete* or *defect-implant* pair for training often fail to generalize to large and complex cranial defects in the test set, since the synthetic defects used during training have different distributions to the real clinical defects during evaluation. One popular solution to this problem is intensive data augmentation: augment the defects [26, 43] and/or augment the skull images [32, 38]. While data augmentation has shown to be effective to the generalization problem, the computational cost is substantially increased. Besides, patient-specific cranial defects show considerable variations among individuals, and it is unlikely to cover every possible defect pattern through augmentation. The SSM-based approach can circumvent the defect-related generalization problem, as an SSM relies only on the complete skulls for training. Therefore, an SSM is insensitive to the changes in defect patterns in the test cases. One factor affecting the performance of an SSM is the registration accuracy. Since human craniums are structurally simple and topologically stable among individuals compared to the defects and facial bones, precise registration among different craniums is highly achievable. Therefore, an SSM or simply a shape warping-based approach is often sufficient for the cranial defect reconstruction task.

## Conclusion

As an alternative to mesh-based SSM (i.e., point distribution model), we demonstrate in this paper that an SSM can be built directly on (volumetric) skull images represented as binary voxel grids. We evaluate the SSM-based method on three cranial defect reconstruction tasks, demonstrating the effectiveness and advantages of the methods on large and complex cranial defects, compared to learning-based approaches. Besides, the SSM-based methods are not dependent on large quantities of training data as deep learning-based approaches, making the proposed method highly scalable and applicable in a clinical setting.

There are two main limitations of the current method that remain to be addressed in future work: (1) When the target and template skulls are not well aligned due to registration errors, non-trivial manual post-processing, as shown in Fig. 4, is required. Poor registration can also cause discontinuities or incompleteness at the junction of the implant and skull surface (e.g., Fig. 6B, C), which undermines the clinical feasibility of the reconstructed implants. Future work could adopt more advanced registration or surface extrapolation methods, as discussed in [41, 42], to further improve the quality of implant reconstruction. (2) Combining the advantages of both SSM and deep learning has not been explored in current work, which should be investigated in the future. SSM-based methods are robust to defect variations and can generate acceptable results stably for large and complex defects, while deep learning-based approaches can leverage large quantities of data to learn more anatomically plausible reconstructions. Integrating deep learning in SSM workflows could potentially improve both aspects.

## Data Availability

All data and codes used in this study are publicly available at https://github.com/Jianningli/ssm and https://doi.org/10.6084/m9.figshare.19328816.v3.

## Footnotes

1 Note that we compute $\mathbf {\Phi }$ explicitly based on Eq. (5)

2 Between two volumes of the same dimension.

3 https://autoimplant2021.grand-challenge.org/

4 Except the last two cases from Task 2.

5 [27, 39] did not report bDSC and HD95. We calculate the two metrics in this paper based on the prediction files (implants) from [27, 39].

## References

[1] Egger J, Pepe A, Gsaxner C, Jin Y, Li J, Kern R (2021) Deep learning-a first meta-survey of selected reviews across scientific disciplines, their commonalities, challenges and research impact. PeerJ Computer Science 7:e773 34901429 10.7717/peerj-cs.773 PMC8627237

[2] Cootes TF, Taylor CJ (1992) Active shape models-‘smart snakes’. In: BMVC92, Springer, pp 266–275

[3] Sclaroff S, Isidoro J (1998) Active blobs. In: Sixth International Conference on Computer Vision (IEEE Cat. No. 98CH36271), IEEE, pp 1146–1153

[4] Cootes TF, Edwards GJ, Taylor CJ (2001) Active appearance models. IEEE Transactions on pattern analysis and machine intelligence 23(6):681–685

[5] Semper-Hogg W, et al. (2017) Virtual reconstruction of midface defects using statistical shape models. Journal of cranio-maxillo-facial surgery : official publication of the European Association for Cranio-Maxillo-Facial Surgery 45(4):461–466 28202219 10.1016/j.jcms.2016.12.020

[6] Fuessinger MA, et al. (2019) Virtual reconstruction of bilateral midfacial defects by using statistical shape modeling. Journal of Cranio-maxillofacial Surgery 47:1054–1059 30982558 10.1016/j.jcms.2019.03.027

[7] Fuessinger MA, et al. (2017) Planning of skull reconstruction based on a statistical shape model combined with geometric morphometrics. International Journal of Computer Assisted Radiology and Surgery 13:519–529 29080945 10.1007/s11548-017-1674-6

[8] Lamecker H (2008) Variational and statistical shape modeling for 3d geometry reconstruction. Master’s thesis, Zuse-Institut Berlin

[9] Pimentel P, Szengel A, Ehlke M, Lamecker H, Zachow S, Estacio L, Doenitz C, Ramm H (2020) Automated virtual reconstruction of large skull defects using statistical shape models and generative adversarial networks. In: Cranial Implant Design Challenge, Springer, pp 16–27

[10] Kun Z (2014) Dense correspondence and statistical shape reconstruction of fractured, incomplete skulls. Master’s thesis, National University of Singapore

[11] Dworzak J, Lamecker H, von Berg J, Klinder T, Lorenz C, Kainmüller D, Seim H, Hege HC, Zachow S (2010) 3d reconstruction of the human rib cage from 2d projection images using a statistical shape model. International journal of computer assisted radiology and surgery 5(2):111–124 20033504 10.1007/s11548-009-0390-2

[12] Kainmueller D, Lamecker H, Zachow S, Hege HC (2009) An articulated statistical shape model for accurate hip joint segmentation. In: 2009 Annual International Conference of the IEEE Engineering in Medicine and Biology Society, IEEE, pp 6345–6351 10.1109/IEMBS.2009.5333269 19964159

[13] Lamecker H, Lange T, Seebass M (2002) A statistical shape model for the liver. In: International conference on medical image computing and computer-assisted intervention, Springer, pp 421–427

[14] Heimann T, Meinzer HP (2009) Statistical shape models for 3d medical image segmentation: a review. Medical image analysis 13(4):543–563 19525140 10.1016/j.media.2009.05.004

[15] Besl PJ, McKay ND (1992) Method for registration of 3-d shapes. In: Sensor fusion IV: control paradigms and data structures, Spie, vol 1611, pp 586–606

[16] Ronneberger O, Fischer P, Brox T (2015) U-net: Convolutional networks for biomedical image segmentation. In: International Conference on Medical image computing and computer-assisted intervention, Springer, pp 234–241

[17] Zhou Z, Rahman Siddiquee MM, Tajbakhsh N, Liang J (2018) Unet++: A nested u-net architecture for medical image segmentation. In: Deep learning in medical image analysis and multimodal learning for clinical decision support, Springer, pp 3–11 10.1007/978-3-030-00889-5_1 PMC7329239 32613207

[18] Li X, Chen H, Qi X, Dou Q, Fu CW, Heng PA (2018) H-denseunet: hybrid densely connected unet for liver and tumor segmentation from ct volumes. IEEE transactions on medical imaging 37(12):2663–2674 29994201 10.1109/TMI.2018.2845918

[19] Milletari F, Navab N, Ahmadi SA (2016) V-net: Fully convolutional neural networks for volumetric medical image segmentation. In: 2016 fourth international conference on 3D vision (3DV), IEEE, pp 565–571

[20] Pepe A, Li J, Rolf-Pissarczyk M, Gsaxner C, Chen X, Holzapfel GA, Egger J (2020) Detection, segmentation, simulation and visualization of aortic dissections: A review. Medical image analysis 65:101773 32738647 10.1016/j.media.2020.101773

[21] Reyneke C, Thusini X, Douglas T, Vetter T, Mutsvangwa T (2018) Construction and validation of image-based statistical shape and intensity models of bone. In: 2018 3rd Biennial South African Biomedical Engineering Conference (SAIBMEC), IEEE, pp 1–4

[22] Grauman K, Shakhnarovich G, Darrell T (2003) Inferring 3d structure with a statistical image-based shape model. In: ICCV, vol 3, p 641

[23] Bharath K, Kurtek S, Rao A, Baladandayuthapani V (2018) Radiologic image-based statistical shape analysis of brain tumours. Journal of the Royal Statistical Society: Series C (Applied Statistics) 67(5):1357–1378 30420787 10.1111/rssc.12272 PMC6225782

[24] Li J, Gsaxner C, Pepe A, Morais A, Alves V, von Campe G, Wallner J, Egger J (2021b) Synthetic skull bone defects for automatic patient-specific craniofacial implant design. Scientific Data 8(1):1–8 33514740 10.1038/s41597-021-00806-0 PMC7846796

[25] Morais A, Egger J, Alves V (2019) Automated computer-aided design of cranial implants using a deep volumetric convolutional denoising autoencoder. In: World Conference on Information Systems and Technologies, Springer, pp 151–160

[26] Li J, von Campe G, Pepe A, Gsaxner C, Wang E, Chen X, Zefferer U, Tödtling M, Krall M, Deutschmann H, et al. (2021a) Automatic skull defect restoration and cranial implant generation for cranioplasty. Medical Image Analysis p 102171 10.1016/j.media.2021.102171 34340106

[27] Li J, Pepe A, Gsaxner C, von Campe G, Egger J (2020) A baseline approach for autoimplant: the miccai 2020 cranial implant design challenge. arXiv preprint arXiv:2006.12449

[28] Li J, Pimentel P, Szengel A, Ehlke M, Lamecker H, Zachow S, Estacio L, Doenitz C, Ramm H, Shi H, et al. (2021e) Autoimplant 2020-first miccai challenge on automatic cranial implant design. IEEE Transactions on Medical Imaging 10.1109/TMI.2021.3077047 33939608

[29] Kodym O, Španěl M, Herout A (2021a) Deep learning for cranioplasty in clinical practice: Going from synthetic to real patient data. Computers in Biology and Medicine 137:104766 34425418 10.1016/j.compbiomed.2021.104766

[30] Ellis DG, Alvarez CM, Aizenberg MR (2021) Qualitative criteria for feasible cranial implant designs. In: Cranial Implant Design Challenge, Springer, pp 8–18

[31] Mahdi H, Clement A, Kim E, Fishman Z, Whyne CM, Mainprize JG, Hardisty MR (2021) A u-net based system for cranial implant design with pre-processing and learned implant filtering. In: Cranial Implant Design Challenge, Springer, pp 63–79

[32] Wodzinski M, Daniol M, Hemmerling D (2021) Improving the automatic cranial implant design in cranioplasty by linking different datasets. In: Cranial Implant Design Challenge, Springer, pp 29–44

[33] Peason K (1901) On lines and planes of closest fit to systems of point in space. Philosophical Magazine 2(11):559–572

[34] Kraevoy V, Sheffer A (2005) Template-based mesh completion. In: Symposium on Geometry Processing, Citeseer, vol 385, pp 13–22

[35] Yu L, Li J, Egger J (2021) Pca-skull: 3d skull shape modelling using principal component analysis. In: Cranial Implant Design Challenge, Springer, pp 105–115

[36] Li J, Ellis DG, Kodym O, Rauschenbach L, Rieß C, Sure U, Wrede KH, Alvarez CM, Wodzinski M, Daniol M, et al. (2023) Towards clinical applicability and computational efficiency in automatic cranial implant design: An overview of the autoimplant 2021 cranial implant design challenge. Medical Image Analysis p 102865 10.1016/j.media.2023.102865 37331241

[37] Li J, Krall M, Trummer F, Memon AR, Pepe A, Gsaxner C, Jin Y, Chen X, Deutschmann H, Zefferer U, et al. (2021c) Mug500+: Database of 500 high-resolution healthy human skulls and 29 craniotomy skulls and implants. Data in Brief 39:107524 34815988 10.1016/j.dib.2021.107524 PMC8591340

[38] Ellis DG, Aizenberg MR (2020) Deep learning using augmentation via registration: 1st place solution to the autoimplant 2020 challenge. In: Cranial Implant Design Challenge, Springer, pp 47–55

[39] Li J, Pepe A, Gsaxner C, Jin Y, Egger J (2021d) Learning to rearrange voxels in binary segmentation masks for smooth manifold triangulation. arXiv preprint arXiv:2108.05269

[40] Egger J, Kapur T, Fedorov A, Pieper S, Miller JV, Veeraraghavan H, Freisleben B, Golby AJ, Nimsky C, Kikinis R (2013) Gbm volumetry using the 3d slicer medical image computing platform. Scientific reports 3(1):1–7 10.1038/srep01364 PMC3586703 23455483

[41] Grupp RB, Chiang H, Otake Y, Murphy RJ, Gordon CR, Armand M, Taylor RH (2015) Smooth extrapolation of unknown anatomy via statistical shape models. In: Medical Imaging 2015: Image-Guided Procedures, Robotic Interventions, and Modeling, SPIE, vol 9415, pp 518–527

[42] Grupp R, Otake Y, Murphy R, Parvizi J, Armand M, Taylor R (2016) Pelvis surface estimation from partial ct for computer-aided pelvic osteotomies. In: Orthopaedic Proceedings, Bone & Joint, vol 98, pp 55–55

[43] Kodym O, Španel M, Herout A (2021b) Skullbreak dataset: An open dataset for training and validation of skull reconstruction models

